# Supplementary material for: Discovery of a Ni2+-dependent heterohexameric metformin hydrolase
Source: Nat Commun. 2024 Jul 20;15:6121. doi: 10.1038/s41467-024-50409-7 (PMC11271267; doi:10.1038/s41467-024-50409-7)
Supplement: Supplementary file 1 — Supplementary Information [file 41467_2024_50409_MOESM1_ESM.pdf]

SUPPLEMENTARY INFORMATION FOR

Discovery of a Ni^{2+}-dependent heterohexameric metformin hydrolase

Tao Li[1], Zhi-Jing Xu[1], Shu-Ting Zhang[1], Jia Xu[1], Piaopiao Pan[1], Ning-Yi Zhou[1]*

[1]State Key Laboratory of Microbial Metabolism, Joint International Research Laboratory of Metabolic and Developmental Sciences, and School of Life Sciences and Biotechnology, Shanghai Jiao Tong University, 200240 Shanghai, China

*Correspondence to Ning-Yi Zhou, Email: ningyi.zhou@sjtu.edu.cn

Contents

Supplementary Figures

Supplementary Figure 1. A conserved gene cluster is present in metformin utilizers.

Supplementary Figure 2. Additional characterization of MetCaCb.

Supplementary Figure 3. Overall structure of the MetCaCb complex.

Supplementary Figure 4. Cryo-EM analysis of metformin hydrolase MetCaCb from strain NyZ550.

Supplementary Figure 5. Cryo-EM micrographs and maps of the MetCaCb complex.

Supplementary Figure 6. Structural differences of MetCa and MetCb.

Supplementary Figure 7. Active-site architecture of MetCaCb.

Supplementary Figure 8. Sequence similarity network (SSN) of the MetCa and MetCb homologs.

Supplementary Tables

Supplementary Table 1. Kinetic parameters of MetCaCb and its variants for metformin or 1-methylbiguanide.

Supplementary Table 2. X-ray diffraction data collection and refinement statistics.

Supplementary Table 3. Cryo-EM data statistics.

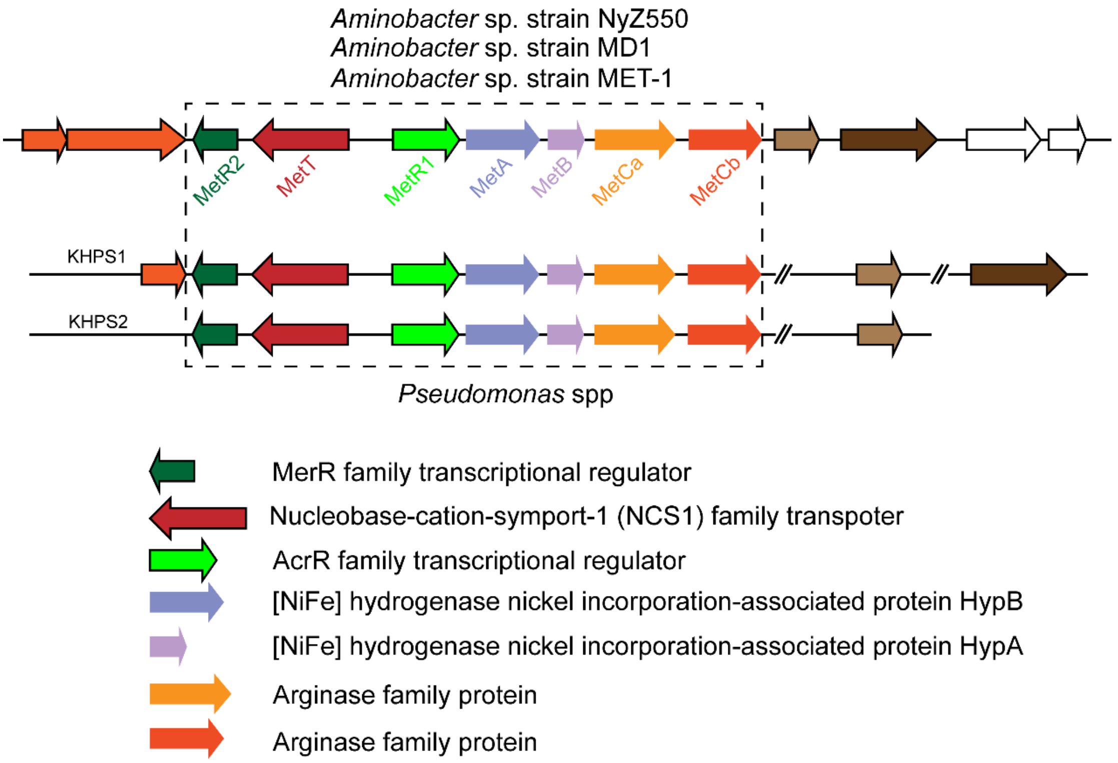

Supplementary Figure 1. A conserved gene cluster is present in metformin utilizers. Metformin degraders include *Aminobacter* strains NyZ550, MD1 and MET-1, as well as *Pseudomonas* strains KHPS1 and KHPS2. The boxed gene cluster was shared by these metformin degraders, and consisted of seven putative genes encoding a MerR family regulatory protein, a nucleobase-cation-symport-1 (NCS1)-like importer, an AcrR-like regulatory protein, two Ni/Fe hydrogenase nickel incorporation-associated proteins (HypA and HypB) and two arginase family proteins.

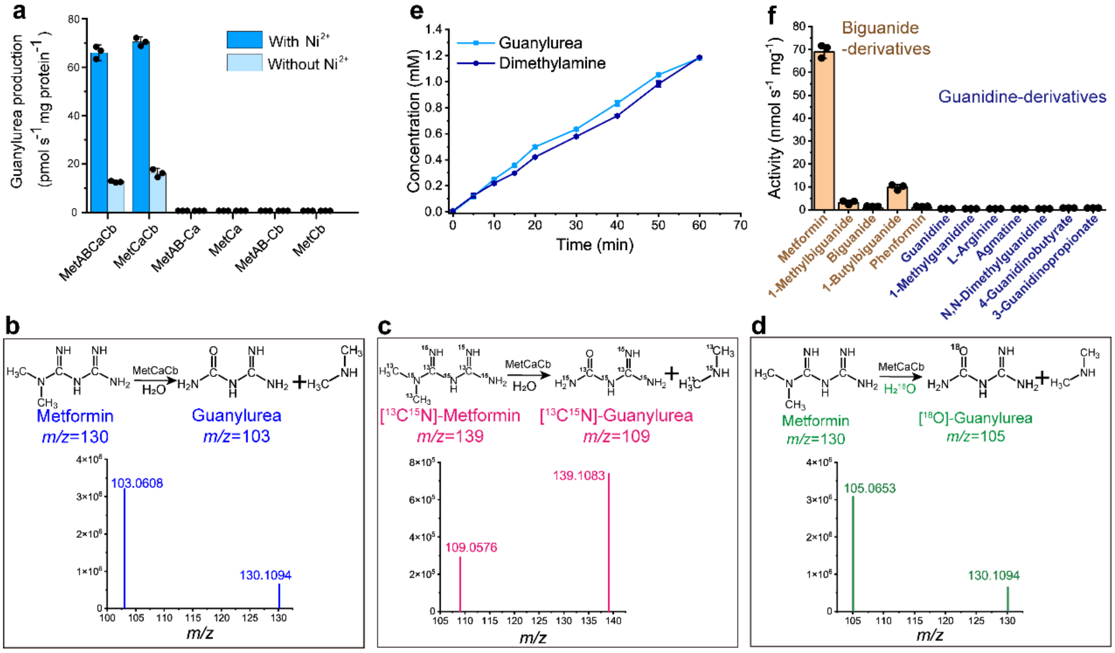

Supplementary Figure 2. Additional characterization of MetCaCb. (a) Conversion of metformin by cell extracts of *E. coli* expressing different combinations of MetABCaCb in the presence or absence of 200 μM nickel ion. Data are presented as mean values ± SD (n = 3 technical replicates). **(b)** LC-MS identification of the product from the hydrolysis of metformin by MetCaCb. **(c)** LC-MS identification of the product from the hydrolysis of $^{13}C^{15}N$-labelled metformin by MetCaCb. **(d)** LC-MS identification of the product from the hydrolysis of metformin by MetCaCb performed in an $H_2{}^{18}O$ solution. **(e)** A representative result of the time-dependent production of guanylurea and dimethylamine from a standard metformin hydrolase assay. The experiment was repeated three times with similar results. **(f)** Hydrolase activity of MetCaCb toward various substrates at a concentration of 10 mM. For biguanide and its derivatives (brown font), the activity tests were based on the production of guanylurea, and for the guanidinium moiety-containing substrates (red font), the activity tests were based on the production of urea. The data are presented as mean values ± SD (n = 3 biological independent replicates). Source data are provided as a Source Data file.

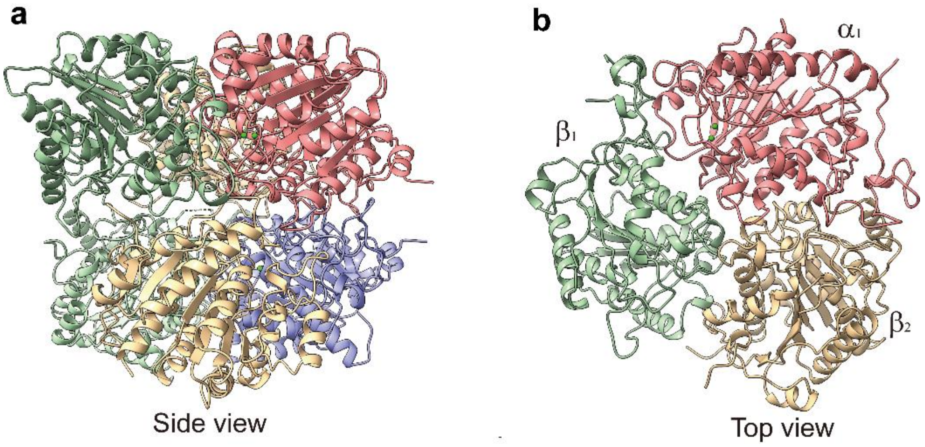

Supplementary Figure 3. Overall structure of the MetCaCb complex. (a) Side view of a cartoon representation of the MetCaCb hexamer. **(b)** Top view of the trimer consisting of one MetCa (claret) and two MetCb (yellow and cyan). Metal ions are shown as green sphere.

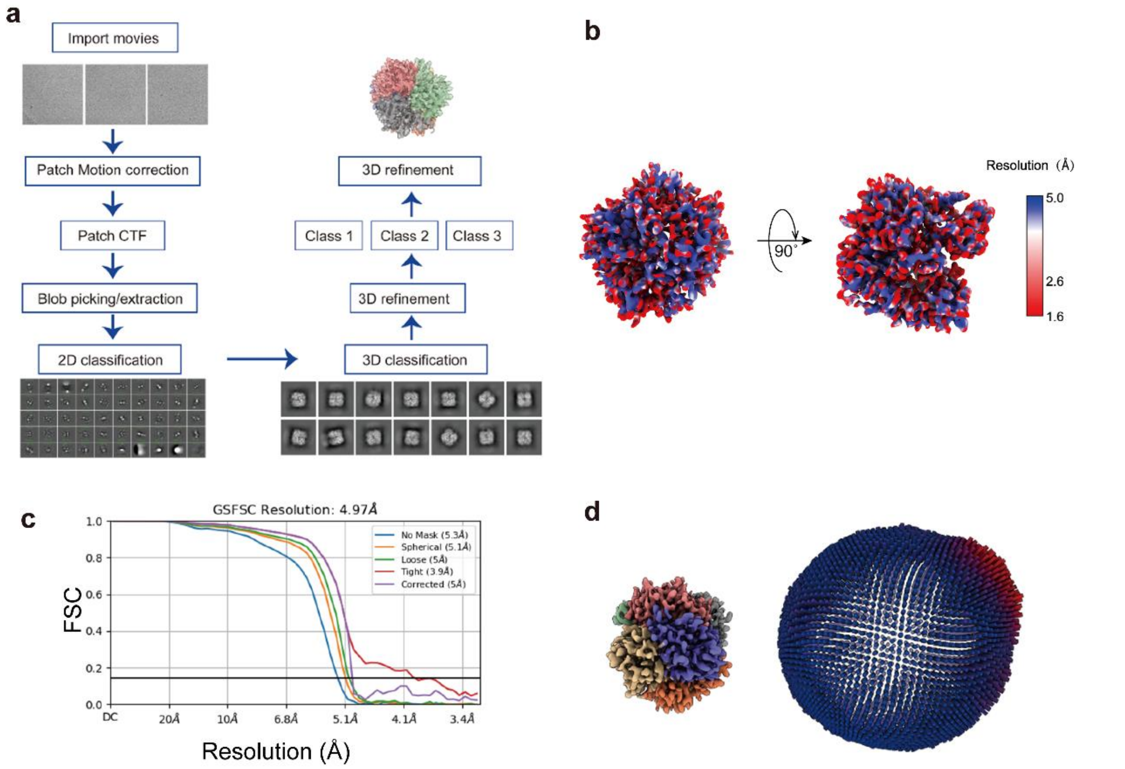

Supplementary Figure 4. Cryo-EM analysis of metformin hydrolase MetCaCb from strain NyZ550. (a) The cryo-EM data processing workflow for MetCaCb. **(b)** Map of MetCaCb cryo-EM density colored by local resolution. **(c)** A global resolution estimate with Fourier Shell Correlation (FSC) cut-off. The resolution at which the gold-standard FSC curve drops below the 0.143 threshold is indicated. **(d)** A representation of the angular distribution of particles used in the final reconstruction.

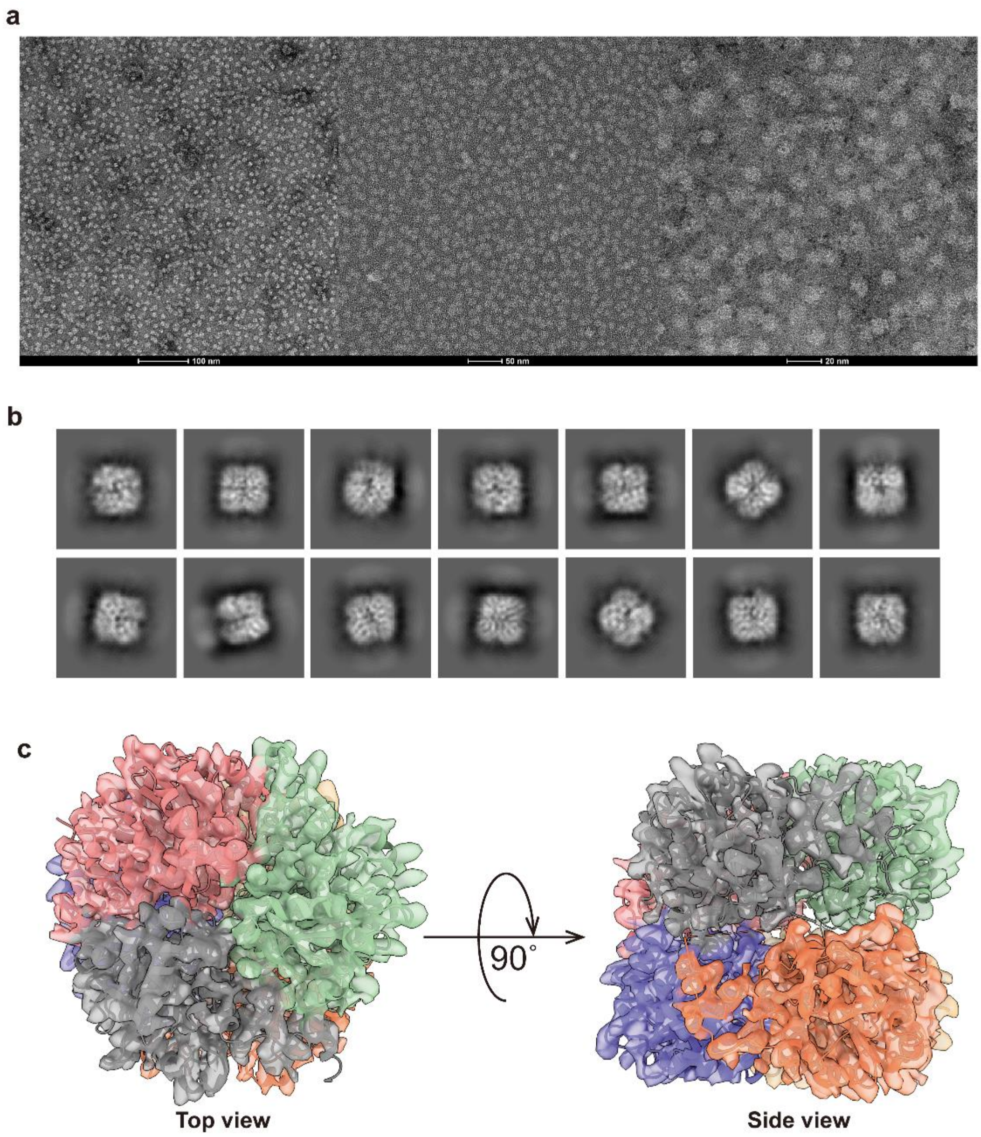

Supplementary Figure 5. Cryo-EM micrographs and maps of the MetCaCb complex. (a) Representative cryoEM micrographs of MetCaCb. Scale bars 200-1,000 Å. **(b)** Representative 2D classes of MetCaCb showing the views of the protein complex. **(c)** Superimposition of the crystal structural model of MetCaCb hexamer on the cryoEM map of MetCaCb. Fitting the crystal structure model in cryoEM map was prepared using UCSF CHIMERA.

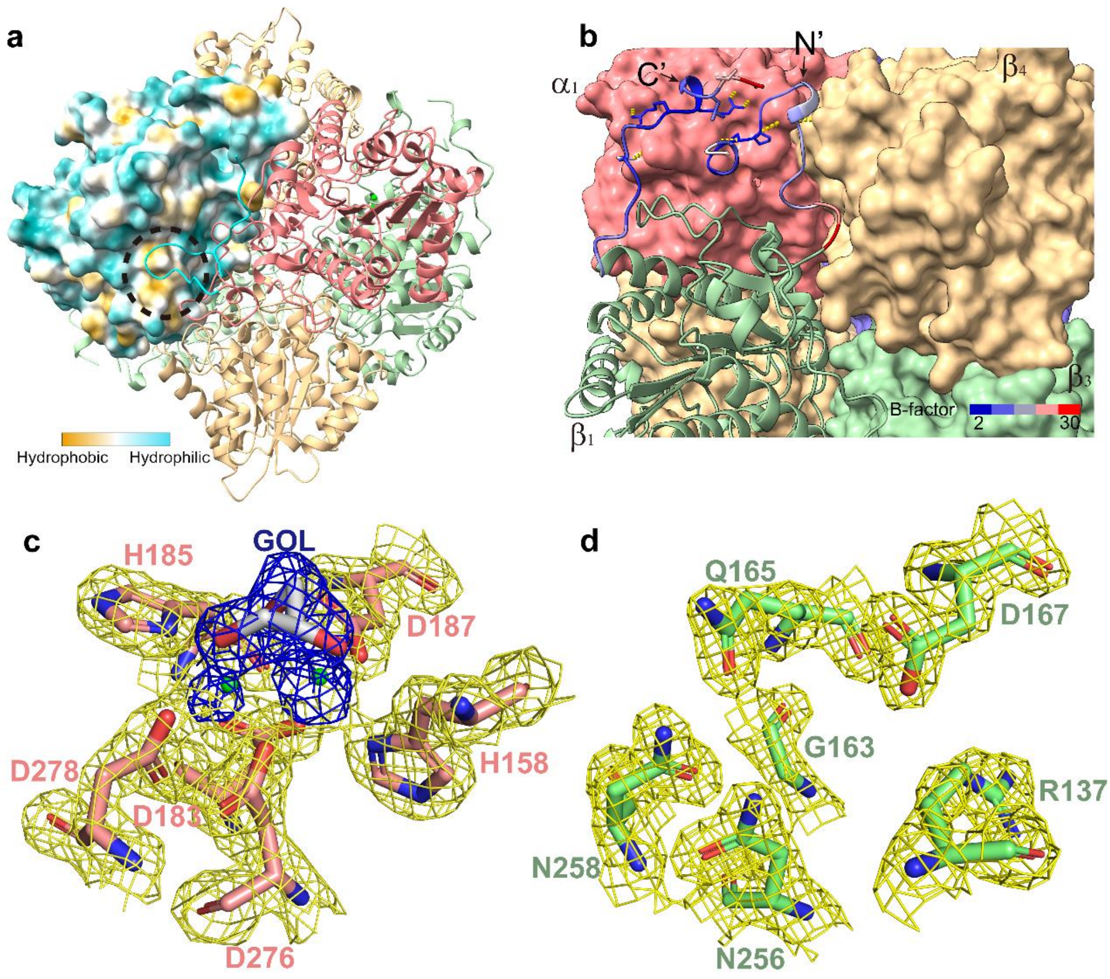

Supplementary Figure 6. Structural differences of MetCa and MetCb. (a) The N-terminal loop structure of MetCa. The circled region shows the interface between two MetCa molecules The N-terminal loop of MetCa binds to a hydrophobic region of the adjacent MetCa. **(b)** Schematic drawing of the N- and C-terminal tails of MetCb. The B-factors of the tail region are indicated by different color and the polar interaction is showed by yellow dash line. The inset enlarged detail shows the interactions between the C-terminal loop of MetCb and MetCa. **(c)** Omit map shows the putative di-metal active site of MetCa. The metal ions are shown as green sphere and the residues coordinating them are shown as sticks. **(d)** Omit map show the corresponding active site region of MetCb compared to MetCa.

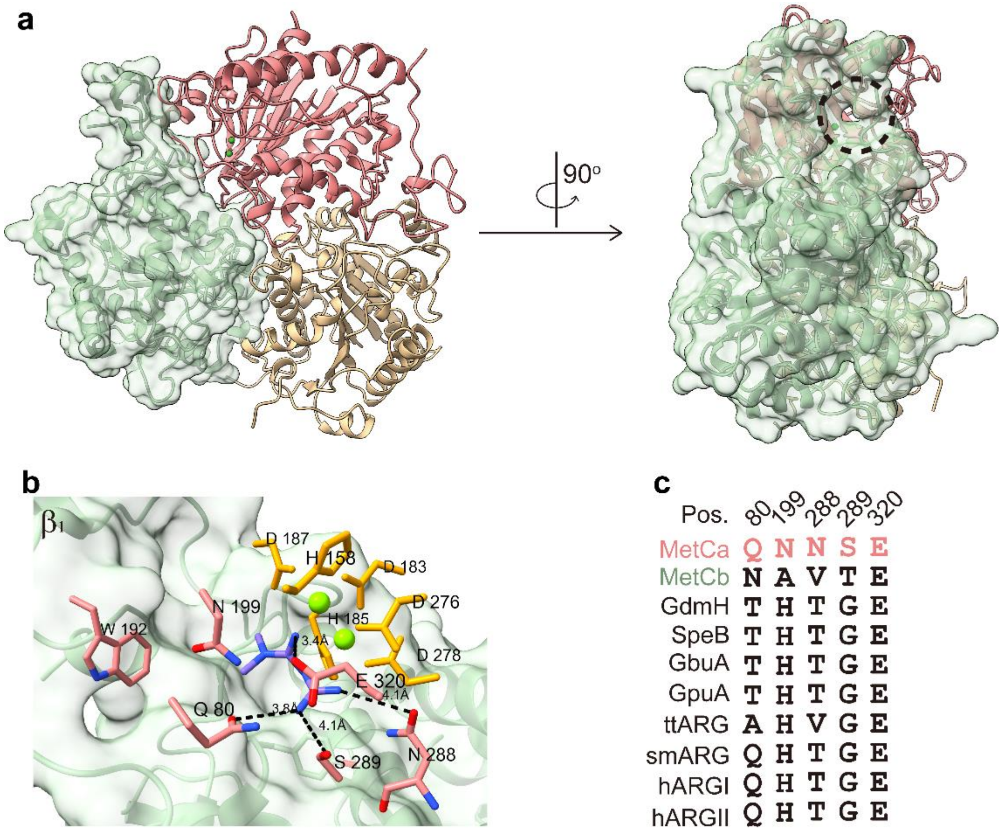

Supplementary Figure 7. Active-site architecture of MetCaCb. **(a)** Schematic drawing of that the active site cavity within the head domain of MetCa is capped by the tail domain of a MetCb subunit. The hollowed-out structure of MetCb (dotted circle) connects the substrate pocket in MetCa. **(b)** Schematic drawing of the active site of MetCa with a metformin molecule (purple stick) docked into it. The capped MetCb is shown as surface representation. **(c)** Sequence alignment of the noncoordinating active site residues of MetCaCb with representative sequences of arginase family proteins. The sequences used included guanidine hydrolase (GdmH), agmatinase (SpeB), guanidinobutyrase (GbuA), guanidinopropionase (GpuA), arginase from *Thermus thermophilus* (ttARG), human arginases I and II (hARGI and hARGII), and arginase from *Schistosoma mansoni* (smARG).

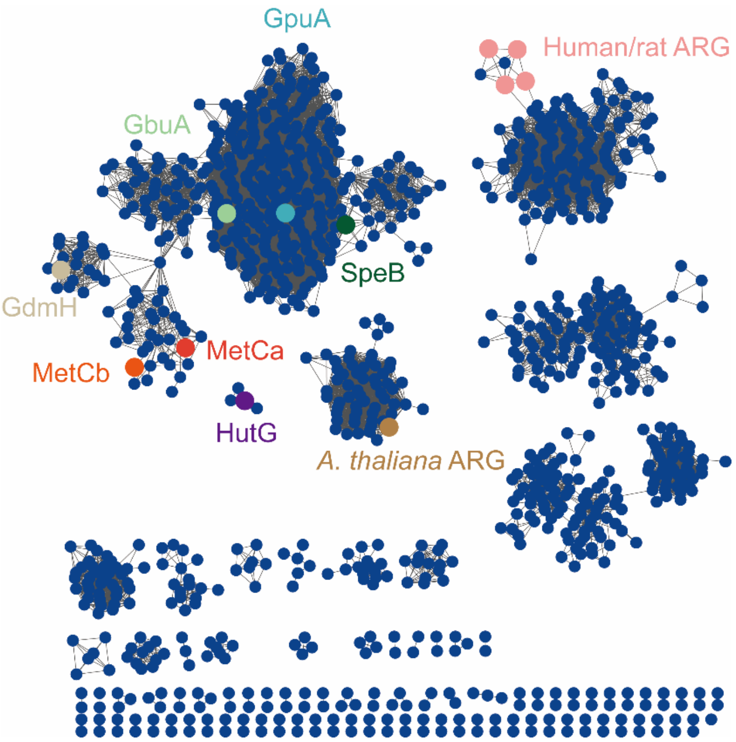

Supplementary Figure 8. Sequence similarity network (SSN) of the MetCa and MetCb homologs. MetCa and MetCb homologous sequences were retrieved from the UniRef90 database using a minimal sequence identity of 25% with the query sequences. The resultant hits were mixed and subjected to filtration by CD-HIT with a cutoff value of 65%, resulting in a total of 1097 sequences. The SSN analysis was carried out by EFI-EST (https://efi.igb.illinois.edu/efi-est/) using the filtered sequences together with other characterized enzymes from arginase/agmatinase family proteins. The visualized clusters were generated from a result using a cutoff alignment score of 60. GdmH: guanidine hydrolase; SpeB: agmatinase; GbuA: guanidinobutyrase; GpuA: guanidinopropionase; HutG: formimidoylglutamase; ARG: arginase.

Supplementary Table 1. Kinetic parameters of MetCaCb and its variants for metformin or 1-methylbiguanide.

Description	K_m (mM)	V_{max} (nM s^{-1})	K_{cat} (s^{-1})	K_{cat}/K_m (mM^{-1} s^{-1})
WT[a], Metformin, pH 9.0	6.84±0.49	815.40±17.29	12.83	1.88
WT, Metformin, pH 10.0	6.42±0.98	453.50±19.30	7.13	1.11
WT, 1-Methylbiguanide, pH 9.0	158.20±24.70	307.60±22.97	4.84	0.03
D183A[b]	ND[c]	ND	ND	ND
H185A	ND	ND	ND	ND
D187A	12.34±2.56	0.59±0.03	0.01	0.0008
D276A	127.10±50.95	2.42±0.52	0.04	0.0003
D278A	ND	ND	ND	ND
N199A	ND	ND	ND	ND
N199H	18.96±4.69	5.20±0.59	0.23	0.01
S289A	15.45±1.51	4.27±0.99	0.07	0.0043
E320A	ND	ND	ND	ND

[a]wild type MetCaCb.

[b]activity of all the MetCaCb variants were measured with the standard metformin hydrolase activity assays.

[c]no detectable activity was observed.

The data are presented as mean values ± SD (n = 2 or 3 biological independent replicates). Source data are provided as a Source Data file.

Supplementary Table 2. X-ray diffraction data collection and refinement statistics

	MetCaCb
**Data collection**	
Space group	$P2_12_12_1$
Cell dimensions	
a, *b*, *c* (Å)	90.43, 152.52, 173.57
α, β, γ (°)	90, 90, 90
Resolution (Å)	39.47-1.84 (1.88-1.84)[a]
R_{merge}	0.119 (0.443)
CC (1/2)	0.995 (0.893)
$I / \sigma I$	12.7 (3.5)
Completeness (%)	100 (99.4)
Redundancy	10.0 (5.7)
**Refinement**	
Resolution (Å)	39.47-1.84
No. reflections	205966
R_{work} / R_{free}	0.153/0.182
No. atoms (occupancy sum)	
Protein	15355 (15355)
Ligand/ion	16 (16)
Water	1576 (1576)
B-factors	
Protein	24.31
Ligand/ion	29.80
Water	32.91
R.m.s. deviations	
Bond lengths (Å)	0.02
Bond angles (°)	1.48
Ramachandran	
Favoured (%)	97.42
Outliers (%)	0.00

[a]Values in parentheses are for highest-resolution shell.

Supplementary Table 3. Cryo-EM data statistics

	MetCaCb
Magnification	92,000
Voltage (kV)	200
Grid type	Quantifoil 1.2/1.3
Microscope	Thermo Scientific Glacios transmission electron microscope
Recording mode	Single particle
Dose rate (e–/Å^2/s)	38.36
Total dose (e/Å)	39.89
Total frames	16
Number of micrographs used	1068
Exposure time (s)	2.54
Defocus range (µm)	−1.5 to −2.5
Pixel size (Å)	1.6
Symmetry imposed	C1
Initial particle images (no.)	2,052,983
Final particle images (no.)	312,045
Map resolution (Å)	~5.0
FSC threshold	0.143
