# Supplementary material for: Discovery of a Ni2+-dependent heterohexameric metformin hydrolase
Source: Nat Commun. 2024 Jul 20;15:6121. doi: 10.1038/s41467-024-50409-7 (PMC11271267; doi:10.1038/s41467-024-50409-7)
Supplement: Supplementary file 2 — Reporting Summary [file 41467_2024_50409_MOESM2_ESM.pdf]

Corresponding author(s): Ning-Yi Zhou
Last updated by author(s): Jun 17, 2024

Reporting Summary

Nature Portfolio wishes to improve the reproducibility of the work that we publish. This form provides structure for consistency and transparency in reporting. For further information on Nature Portfolio policies, see our Editorial Policies and the Editorial Policy Checklist.

Statistics

For all statistical analyses, confirm that the following items are present in the figure legend, table legend, main text, or Methods section.

n/a	Confirmed	
☐	☒	The exact sample size (*n*) for each experimental group/condition, given as a discrete number and unit of measurement
☐	☒	A statement on whether measurements were taken from distinct samples or whether the same sample was measured repeatedly
☒	☐	The statistical test(s) used AND whether they are one- or two-sided<br>*Only common tests should be described solely by name; describe more complex techniques in the Methods section.*
☒	☐	A description of all covariates tested
☒	☐	A description of any assumptions or corrections, such as tests of normality and adjustment for multiple comparisons
☐	☒	A full description of the statistical parameters including central tendency (e.g. means) or other basic estimates (e.g. regression coefficient) AND variation (e.g. standard deviation) or associated estimates of uncertainty (e.g. confidence intervals)
☒	☐	For null hypothesis testing, the test statistic (e.g. *F*, *t*, *r*) with confidence intervals, effect sizes, degrees of freedom and *P* value noted<br>*Give P values as exact values whenever suitable.*
☒	☐	For Bayesian analysis, information on the choice of priors and Markov chain Monte Carlo settings
☒	☐	For hierarchical and complex designs, identification of the appropriate level for tests and full reporting of outcomes
☒	☐	Estimates of effect sizes (e.g. Cohen's *d*, Pearson's *r*), indicating how they were calculated

Our web collection on statistics for biologists contains articles on many of the points above.

Software and code

Policy information about availability of computer code

Data collection	Sequence similarity network: Consurf webserver (https://consurf.tau.ac.il/), CD-HIT (https://www.bioinformatics.org/cd-hit/), EFI-EST (https://efi.igb.illinois.edu/efi-est/), Cytoscape 3.10.0; Sequence alignment: Cblaster v1.3.18;
Data analysis	lot: Origin 2021b education version; Crystallography: XDS program package (2022 release), PHENIX version 1.20.1_4487 which implanted PHASER, MolProbity, COOT; Docking: AutoDock Vina v1.1.2; CyroEM movie processing: cryoSPARC v3.1; Modelling: ChimeraX version 1.4; Structure prediction: Alphafold2 (https://colab.research.google.com/github/sokrypton/ColabFold/blob/main/AlphaFold2.ipynb#scrollTo=G4yBrceuFbf3); Maximum likelihood phylogenetic tree: MEGA version 11.0.13, visualized with iTOL (https://itol.embl.de/) ; Multiple sequence alignment: MUSCLE implanted in MEGA version 11.0.13. NMR data analysis: TopSpin 4.3.0;

For manuscripts utilizing custom algorithms or software that are central to the research but not yet described in published literature, software must be made available to editors and reviewers. We strongly encourage code deposition in a community repository (e.g. GitHub). See the Nature Portfolio guidelines for submitting code & software for further information.

Data

Policy information about availability of data

All manuscripts must include a data availability statement. This statement should provide the following information, where applicable:

- Accession codes, unique identifiers, or web links for publicly available datasets
- A description of any restrictions on data availability
- For clinical datasets or third party data, please ensure that the statement adheres to our policy

Atomic coordinate and structure factor for the crystal structure of MetCaCb from Aminobacter sp. strain NyZ550 have been deposited to the Protein Data Bank (PDB) under accession number 8X3G . The cryoEM movies and the refined model are available from the corresponding author upon request.

Research involving human participants, their data, or biological material

Policy information about studies with human participants or human data. See also policy information about sex, gender (identity/presentation), and sexual orientation and race, ethnicity and racism.

Reporting on sex and gender	This does not apply to this study
Reporting on race, ethnicity, or other socially relevant groupings	This does not apply to this study
Population characteristics	This does not apply to this study
Recruitment	This does not apply to this study
Ethics oversight	This does not apply to this study

Note that full information on the approval of the study protocol must also be provided in the manuscript.

Field-specific reporting

Please select the one below that is the best fit for your research. If you are not sure, read the appropriate sections before making your selection.

☒ Life sciences ☐ Behavioural & social sciences ☐ Ecological, evolutionary & environmental sciences

For a reference copy of the document with all sections, see nature.com/documents/nr-reporting-summary-flat.pdf

Life sciences study design

All studies must disclose on these points even when the disclosure is negative.

Sample size	Sample sizes were not relevant for this study because no complex statistical method was used or a statistical evaluation of effects between two or more groups.
Data exclusions	No data were excluded.
Replication	Experiments were replicated at least twice; results were consistent throughout the experiments. Furthermore, the reproducibility of the assays was confirmed by including appropriate positive and negative controls.
Randomization	Randomization is not significant for biochemical experiments. The products, catalyzed reactions, and structures were unknown during these assays.
Blinding	The authors were not blinded, because no subjective analyses were performed.

Reporting for specific materials, systems and methods

We require information from authors about some types of materials, experimental systems and methods used in many studies. Here, indicate whether each material, system or method listed is relevant to your study. If you are not sure if a list item applies to your research, read the appropriate section before selecting a response.

Materials & experimental systems

n/a	Involved in the study
☒	☐ Antibodies
☒	☐ Eukaryotic cell lines
☒	☐ Palaeontology and archaeology
☒	☐ Animals and other organisms
☒	☐ Clinical data
☒	☐ Dual use research of concern
☒	☐ Plants

Methods

n/a	Involved in the study
☒	☐ ChIP-seq
☒	☐ Flow cytometry
☒	☐ MRI-based neuroimaging

Plants

Seed stocks

Report on the source of all seed stocks or other plant material used. If applicable, state the seed stock centre and catalogue number. If plant specimens were collected from the field, describe the collection location, date and sampling procedures.

Novel plant genotypes

Describe the methods by which all novel plant genotypes were produced. This includes those generated by transgenic approaches, gene editing, chemical/radiation-based mutagenesis and hybridization. For transgenic lines, describe the transformation method, the number of independent lines analyzed and the generation upon which experiments were performed. For gene-edited lines, describe the editor used, the endogenous sequence targeted for editing, the targeting guide RNA sequence (if applicable) and how the editor was applied.

Authentication

Describe any authentication procedures for each seed stock used or novel genotype generated. Describe any experiments used to assess the effect of a mutation and, where applicable, how potential secondary effects (e.g. second site T-DNA insertions, mosiacism, off-target gene editing) were examined.
