# Supplementary material for: Discovery of a Ni2+-dependent heterohexameric metformin hydrolase
Source: Nat Commun. 2024 Jul 20;15:6121. doi: 10.1038/s41467-024-50409-7 (PMC11271267; doi:10.1038/s41467-024-50409-7)
Supplement: Supplementary file 4 — Description of Additional Supplementary Files [file 41467_2024_50409_MOESM4_ESM.pdf]

**File name: Supplementary Data 1**

Description: The file provides the aligned amino acid sequences of MetCaCb homologous pairs in FASTA style. Sequence length and species information are specified.

**File name: Supplementary Data 2**

Description: The file provides the primer sequences used for amplification of DNA fragments in this study.
